# Could a Dolutegravir-Based Antiretroviral Therapy Lead to Clinical Obesity? A Retrospective Cohort Study Conducted at Hawassa University Comprehensive Specialized Hospital in Hawassa, Sidama, Ethiopia

**DOI:** 10.1155/2022/2965325

**Published:** 2022-05-13

**Authors:** By Aberash Eifa, Worku Ketema

**Affiliations:** ^1^Department of Midwifery, College of Health Sciences, Hawassa University, P.O.Box 1560, Hawassa, Ethiopia; ^2^Department of Pediatrics and Child Health, College of Health Sciences, Hawassa University, P.O.Box 1560, Hawassa, Ethiopia

## Abstract

**Background:**

As of April 2019, the dolutegravir (DTG)-based regimen is replacing the efavirenz-based regimen in Ethiopia, mainly due to its superiority in viral load suppression. However, there is a growing concern about this medication-based regimen, the most serious of which is excessive weight gain. In this study, we looked at weight gain disparities among human immunodeficiency virus/acquired immunodeficiency syndrome (HIV/AIDS) patients on antiretroviral therapy (ART) who have been shifted to tenofovir/lamivudine/dolutegravir (TLD) from a tenofovir/lamivudine/efavirenz (TLE)-based regimen versus those who are maintained on a tenofovir/lamivudine/efavirenz-based regimen.

**Methods:**

A facility-based retrospective observational cohort study was conducted in pursuit of weight change disparities between tenofovir/lamivudine/dolutegravir and tenofovir/lamivudine/efavirenz-based regimens among patients who have attained optimal viral suppression at Hawassa University Comprehensive Specialized Hospital antiretroviral clinic. Chi-square and logistic regression were used as appropriate using an SPSS version 21 program to test the association of specific variables to outcome variables, and a *P* value <0.05 was considered statistically significant.

**Results:**

This study included 422 patients, 211 of whom were switched from tenofovir/lamivudine/efavirenz to tenofovir/lamivudine/dolutegravir and the remaining were who kept on a tenofovir/lamivudine/efavirenz-based regimen. Patients on a tenofovir/lamivudine/dolutegravir-based regimen had a mean weight gain of 3.88 ± 2.021 kg in one year compared to those on TLE (2.26 ± 2.39). In a bivariate analysis, being male was found to protect against unwanted weight gain at COR 0.531 (0.345, 0.816). A current CD4 count of more than 500 has been found to be strongly correlated with weight gain in multivariate analysis at an AOR of 0.315 (0.188, 0.527) at a *P* value ≤0.001.

**Conclusion:**

According to this study, tenofovir/lamivudine/dolutegravir (TLD)-based antiretroviral medication (ART) users are more likely to gain weight, and clinicians should advise them of the risks of weight gain as well as cost-effective ways to prevent weight gain linked to poor health outcomes in these patients. Future investigations should confirm the findings of this study, and more research into the effects of weight gain in these people is required.

## 1. Introduction

Since the discovery of highly active antiretroviral therapy (HAART), the treatment of human immunodeficiency virus/acquired immunodeficiency syndrome (HIV/AIDS) has been revolutionized, as evidenced by a drastically reduced rate of death from HIV/AIDS and a decrease in the occurrence of opportunistic infections [1–3].

Dolutegravir, a recently added integrase inhibitor antiretroviral drug, impairs HIV integrase function and thus prevents HIV insertion into the host cell's DNA [1, 4–6]. It is not uncommon to see a short-term weight gain in HIV/AIDS patients who begin ART, particularly in those with a pretreatment low body mass index (BMI) and significantly low CD4 at the time of treatment. [7–9]. To put it another way, the weight gain following ART initiation was once one of the tools used to assess ART success [7, 10, 11].

Given the significantly increased prevalence of noncommunicable diseases, weight gain reevaluation has exhibited a significant association of weight gain with metabolic syndrome, imperiling HIV/AIDS care yet further [12–14]. A multisite study conducted in the United States of America found that more than half of HIV/AIDS patients on ART for more than three years had clinical obesity, putting them at risk of developing noncommunicable diseases [4, 9, 15]. According to a 2019 WHO meta-analysis, an absolute increase in body weight of 3–5 kg has been ascertained among patients on a DTG-based regimen at about 48 weeks after ART initiation. As a result, individuals on a DTG-based regimen are strongly advised to abide by weight-loss specific suggestions, such as regular exercise [1–4, 9, 16].

Since the first ART, there has been a constant need for the development of better drugs in all areas of pharmacology [3, 12]. As of April 2019, Ethiopia has begun to implement a new ART regimen as a preferred first-line ART based on WHO recommendations, which is a step in the right direction [13]. Instead of the well-known efavirenz-based ART regimen, a dolutegravir-based ART regimen is being used. The main reasons why this new medication was preferred over the existing medication were the good safety profile, rapid suppression of viral load, and high genetic variability for resistance, to mention a few [1, 4, 5]. Implementing the recommended regimen will be critical in the fight against HIV/AIDS, though, since evidence-based advice will dramatically improve coverage and achieve the desired treatment outcome. Despite these advantages, there is growing concern about this new regimen, with many patients complaining of unintended weight gain [1, 3, 6, 14, 17, 18].

The decrease in morbidity and mortality among people living with the human immunodeficiency virus (PLWHA) has been linked to a rise in noncommunicable disease rates, notably metabolic disorders. In recent studies, virologically suppressed people living with HIV (PLWH) who transitioned from old antiretroviral medication (ART) to newer integrase strand transfer inhibitor (INSTI)-based regimens gained weight [1, 19, 20]. Given the potentially serious public health effects that could threaten the national widespread use of dolutegravir, it will be vital to follow the impact of the DTG-based ART regimen on clinical obesity. Because there are no data on these findings in the study area, we looked for significant weight change differences between patients who switched to a DTG-based regimen (TLD group) and those who stayed on an efavirenz-based regimen (TLE group).

## 2. Methods

### 2.1. Study Design and Setting

A retrospective observational cohort study was conducted at Hawassa University's comprehensive specialized hospital (HUCSH), which is located in Hawassa city, the capital of the SNNPR and Sidama region, 273 kilometers south of Addis Ababa. The boundaries of Hawassa city are defined by Lake Hawassa in the east, Oromia in the west, Wondogenet woreda in the north and east, and Shebadeno in the south.

The city government covers a total of 157.2 square meters. Eight subcities and 32 kebeles make up the total area of km^2^. Two public hospitals and nine public health centers are also part of the city government. The Regional Health Bureau built Hawassa University's comprehensive specialized hospital (HUCSH) 16 years ago. The hospital was built with the intention of serving a population of 3.5–5 million people, but it currently serves the whole Sidama, SNNPR, and Oromia regions (HUSCH human recourse office). It is a tertiary hospital with more than 20 departments that provide both clinical and academic services. The ART clinic is one of the clinical services, and there are now roughly 2620 PLWHA on first-line ART, with nearly 55% of them on DTG-based regimens. The research was carried out from January to March 2021, G.C.

### 2.2. Population

#### 2.2.1. Source Population

The source population was all adult HIV patients who were on ART in HUCSH ART clinic.

#### 2.2.2. Study Population

All HIV patients who have been shifted from TLE to TLD and those who have been maintained on TLE have been on a specified regimen for at least one year.

### 2.3. Eligibility Criteria

#### 2.3.1. Inclusion Criteria

All adult AIDS patients who have been on TLE-based regimen for at least 1 year and obtained optimal viral suppression and adult patients who had been shifted to TLD from TLE-based ART regimen, and stayed on TLD for at least 1 year and attained optimal viral load suppression in HURCSH ART clinic.

#### 2.3.2. Exclusion Criteria

AIDS patients who are on ART for less than one year, any patient with deliberate or unintended overdose, age less than 18, weight less than 26 kg, on second-line regimen, patients with unsuppressed viral load have been excluded from the study.

### 2.4. Sample Size

The required sample size was estimated using Epi Info, version 7, statistical software, with the following assumptions: 95% confidence interval, 80% power, and a 1 : 1 ratio of exposed (DTG-based ART regimen) to nonexposed (TLE-based ART regimen). 422 was the final sample size.

### 2.5. Sampling Method

The simple random sampling method was used to collect the data by reviewing the cards for bodyweight in kilograms (kg), body mass index (BMI), and other pertinent information based on the prepared tools.

### 2.6. Study variables

#### 2.6.1. Dependent Variables

Weight change among the currently preferred first-line ART regimens based on national guideline and WHO recommendations (i.e., TLD and TLE).

#### 2.6.2. Independent Variables

1. Sociodemographic variables.
2. Clinical and laboratory state.
3. ART regimens.
4. Drugs used other than ART drugs.
5. Opportunistic infections.
6. Concomitant medical illness other than OI.

### 2.7. Data Collection Procedure and Source of Data

Standardized survey questionnaires were prepared to collect relevant data by reviewing the patient's charts. The charts of adults who were on ART in the HUCSH ART clinic were reviewed based on the prepared standardized questionnaires. Data collection was performed by trained general practitioners who were actively working at HUCSH ART clinic. Two medical doctors who were from another health facility were working as supervisors, and close supervision of the data collectors was being undertaken by the supervisors.

### 2.8. Data Processing, Analysis, and Data Quality Assurance

The collected data were being cross-checked daily for the accuracy of the information. Chi-square and relatives logistic regression were used as appropriate using an SPSS version 21 program to test the association of specific variables to outcome variables, and a *P* value <0.05 was considered statistically significant. Results were presented using texts, tables, pie charts, and bar graphs were interpreted by looking at the proportion in percent and odds ratios with 95% confidence interval. A weight change greater than 5% was taken as a significant weight gain (clinical obesity) [1, 21–23]. A properly designed data collection instrument was used and pretested on 21 patients who were enrolled at the Adare General Hospital ART clinic. A few amendments were made to the flow of the questionnaire. For analysis, the collected data were entered, cleaned, and managed with SPSS version 22.

## 3. Results

In this study, we evaluated 422 HIV/AIDS patients, equally divided into TLD- and TLE-based groups, with a 100% response rate. Across both study groups, the proportions of age, gender, marital status, and educational status were virtually identical (see Table 1).

The majority of the TLD-based groups with more than 5% weight gain were in WHO stage 2 when they started ART, whereas the TLE groups were in WHO stage 3. Lower CD4 counts at the start of ART were found to be associated with significant weight gain in both groups when compared to high CD4 counts, whereas viral load has the opposite relationship (Table 2).

The majority of same-day ART initiations were reported in those with a weight gain greater than 5%, particularly in the TLD group, and the least weight gain was observed in those who began ART within one month of knowing their status (see Table 3).

Despite the fact that a higher proportion of the TLD group is on TB treatment, the median provision of CPT and INH remained nearly comparable between the two groups. Metformin was the most commonly prescribed medication for illnesses other than HIV/AIDS in both groups, followed by combinations of Lasix plus digoxin plus spironolactone. Patients on a TLD-based regimen had a mean weight gain of 3.88 ± 2.021 kg in one year compared to those on TLE (2.26 ± 2.39) (see Table 4).

The TLD group that gained significant weight gain of 5% or more experienced the longest duration of weight gain within 48 weeks of ART initiation (67.0 percent). However, between the two groups, the least weight change was observed within 12 weeks of ART initiation. Diabetes mellitus was the most commonly diagnosed medical disease in both groups, with a higher prevalence in the TLE group (28.6 percent vs. 15.9 percent, respectively). The majority of the study groups experienced adverse drug reactions (ADRs) during treatment, with peripheral neuropathy symptoms such as numbness, tingling, and pain being the most common (in about 21.1 percent of TLD and 10.2 percent of TLE). The advancement of ADR was reported mostly within 12 weeks and above in the TLD group, but within 4 weeks in the TLE analog.

In a bivariate analysis, being male was found to protect against unwanted weight gain at COR 0.531 (0.345, 0.816), whereas having an initial viral load of more than 1000 at the start of ART and starting on TLD were found to be risk factors for unwanted weight gain at COR 3.258 (1.943, 5.466), and COR 2.055 (1.390, 3.039), respectively, at a *P* value less than or equal to 0.2. The multivariate analysis in this study revealed that a current CD4 count greater than 500 is significantly associated with weight gain with an AOR of 0.315 (0.188, 0.527) at a *P* value of ≤0.001 (see Table 5).

## 4. Discussions

In this study, we evaluated 422 HIV/AIDS patients, equally divided into TLD- and TLE-based groups, with a 100% response rate. Across both study groups, the proportions of age, gender, marital status, and educational status were virtually identical. The majority of the TLD-based group was at WHO stage 2 when they began ART, whereas the TLE group was at WHO stage 3. One of the criteria for switching patients from the TLE to the TLD regimen is long-term viral suppression, which has something to do with the WHO clinical stage [3, 18].

The TLE group reported the majority of same-day ART initiation (20.6 percent), while the TLD group had a higher proportion of those who started ART within one week of learning their status. Meticulous workup is required prior to initiating ART, despite the fact that early initiation is the avocation at the moment, particularly as the regimen shifts from TLE-based to TLD-based [1, 3, 18].

Patients on a TLD-based regimen had a mean weight gain of 3.88 ± 2.02 kg in one year compared to those on TLE (2.26 ± 2.39). Because TLD has a faster viral suppression rate than TLE, the former group will gain more weight, in addition to the class-specific weight gain of integrase inhibitors [24–31]. This is almost identical to the study conducted by Kassem et al. [6], Phillips et al. [16], Jamison et al. [32], and Phillips et al. [33]. The WHO recommendation in its latest update, 2019, also warns of the possibility of excessive weight gain of about 3–5 kg in DTG-based regimens. Therefore, our study's findings will be supported further by the WHO recommendation in its latest update [3, 4]. One of the possible mechanisms for DTG-associated weight gain has been proposed as a decrease in energy expenditure following the rapid reduction of viral load following the initiation of a DTG-based regimen [1, 2, 16].

In both groups, diabetes mellitus was the most commonly diagnosed medical condition, particularly in the TLE group (28.6 percent and 15.9 percent, respectively). This could be due to the alarmingly high prevalence of diabetes mellitus in the general population [13, 34]. Metformin was the most commonly prescribed medication for non-AIDS medical indications in this study. This is most likely due to current trends of rising noncommunicable disease prevalence, such as diabetes mellitus, among HIV/AIDS patients in particular, and the general population in general. As a result, it is critical to strictly monitor the patient's serum blood glucose as DTG is reported [3, 18, 35].

The multivariate analysis in this study revealed that a current CD4 count greater than 500 is significantly associated with weight gain with an AOR of 0.315 (0.188, 0.527) at a *P* value of ≤0.001. The literature indicates a short-term weight gain in HIV/AIDS patients who begin ART, particularly those with a low pretreatment BMI and significantly low CD4 at the time of ART initiation [7–9]. As a result, we recommend this population's careful interpretation of weight gain, as not all weight gain is harmful to one's health.

In general, DTG-based ART has been shown to increase HIV/AIDS patients' morbidity and mortality by increasing the likelihood of clinical obesity, and as a consequence, metabolic syndrome. As a result, keeping a watch out for clinical obesity and intervening early is advised.

## 5. The Strength and Limitations

### 5.1. Strength

As far as we know, this is the first study of its kind (there is no single study conducted in our country on this specific topic). It also compares two groups of patients, with the results aiding clinicians in alerting their patients as early as possible before the onset of metabolic syndrome as a consequence of clinical obesity.

### 5.2. Limitations

There are limitations to this research. The fact that this is a retrospective study has its own set of drawbacks, as does the fact that it is limited to a particular hospital. Other individuals on non-efavirenz-based regimens were not included in the study. We believe that more research should be conducted, and that the study findings presented in this report should be considered with care.

## 6. Conclusions

According to this study, tenofovir/lamivudine/dolutegravir (TLD)-based antiretroviral medication (ART) users are more likely to gain weight, and clinicians should advise them on the risks of weight gain as well as cost-effective ways to prevent weight gain linked to poor health outcomes in these patients. Future investigations should confirm the findings of this study, and more research into the effects of weight gain in these people is required.

## Figures and Tables

**Table 1:** Socioeconomic status of HIV/AIDS patients on ART who enrolled in the ART clinic at Hawassa University Comprehensive Specialized Hospital in March 2021.

Variable	Category	Weight change				Total
		<5%		≥5%		
		TLD	TLE	TLD	TLE	
Sex	Male, n (%)	39 (42.9%)	57 (57.6%)	52 (57.1%)	42 (42.4%)	190 (100.0%)
	Female, n (%)	35 (29.2%)	54 (48.2%)	85 (70.8%)	58 (51.8%)	232 (100.0%)
	Total	74 (35.1%)	111 (52.6%)	137 (64.9%)	100 (47.4%)	422 (100.0%)

Marital status	Single	15 (36.6%)	24 (51.1%)	26 (63.4%)	23 (48.9%)	88 (100.0%)
	Married	46 (36.2%)	64 (56.1%)	81 (63.8%)	50 (43.9%)	241 (100.0%)
	Divorced	8 (38.1%)	10 (43.5%)	13 (61.9%)	13 (56.5%)	44 (100.0%)
	Widowed	5 (22.7%)	13 (48.1%)	17 (77.3%)	14 (51.9%)	49 (100.0%)
	Total	74 (35.1%)	111 (52.6%)	137 (64.9%)	100 (47.4%)	422 (100.0%)

Educational status	Illiterates	10 (23.8%)	19 (47.5%)	32 (76.2%)	21 (52.5%)	82 (100.0%)
	Primary	31 (36.0%)	51 (56.7%)	55 (64.0%)	39 (43.3%)	176 (100.0%)
	Secondary	23 (41.8%)	27 (50.0%)	32 (58.2%)	27 (50.0%)	109 (100.0%)
	College and above	10 (35.7%)	14 (51.9%)	18 (64.3%)	13 (48.1%)	55 (100.0%)
	Total	74 (35.1%)	111 (52.6%)	137 (64.9%)	100 (47.4%)	422 (100.0%)

**Table 2:** Initial and current laboratory parameters for weight change among HIV/AIDS patients on ART who enrolled in the ART clinic at Hawassa University Comprehensive Specialized Hospital in March 2021.

Variables	Category	Weight change				Total
		<5		≥5		
		TLD	TLE	TLD	TLE	
WHO stage	I	22	23	30	22	97
		42.3%	51.1%	57.7%	48.9%	100.0%
	II	21	25	51	21	118
		29.2%	54.3%	70.8%	45.7%	100.0%
	III	24	55	47	51	177
		33.8%	51.9%	66.2%	48.1%	100.0%
	IV	7	8	9	6	30
		43.8%	57.1%	56.3%	42.9%	100.0%
	Total	74	111	137	100	422
		35.1%	52.6%	64.9%	47.4%	100.0%

Initial CD4 count	CD4 <500	40	74	81	64	145
		33.1%	53.6%	66.9%	46.4%	56.0%
	CD4 ≥500	34	37	56	36	92
		37.8%	50.7%	62.2%	49.3%	56.4%
	Total	74	111	137	100	237
		35.1%	52.6%	64.9%	47.4%	56.2%

Current CD4	CD4 <500	34	96	60	57	213
		56.7%	62.7%	100.0%	37.3%	100.0%
	CD4 ≥500	40	15	151	43	209
		26.5%	25.9%	100.0%	74.1%	100.0%
	Total	74	111	211	100	422
		35.1%	52.6%	100.0%	47.4%	100.0%

Viral load after 1 year	Viral load ≥1000	18	35	12	14	79
		60.0%	71.4%	40.0%	28.6%	100.0%
	Viral load <1000	56	76	125	86	343
		30.9%	46.9%	69.1%	53.1%	100.0%
	Total	74	111	137	100	422
		35.1%	52.6%	64.9%	47.4%	

**Table 3:** Timing of ART initiation and weight changes in HIV/AIDS patients on ART who enrolled in the ART clinic at Hawassa University Comprehensive Specialized Hospital in March 2021.

Variable	Category	Weight change				Total
		<5%		≥5%		
		TLD	TLE	TLD	TLE	
Time of diagnosis and ART start	The same-day ART initiation	28	45	42	42	157
		40.0%	51.7%	60.0%	48.3%	100.0%
	Within 1 wk. ART initiation	37	51	74	39	201
		33.3%	56.7%	66.7%	43.3%	100.0%
	Within 2 wks. ART initiation	4	6	10	11	31
		28.6%	35.3%	71.4%	64.7%	100.0%
	Within 1 month	5	9	11	8	33
		31.3%	52.9%	68.8%	47.1%	100.0%
	Total		111		100	422
			52.6%		47.4%	100.0%

**Table 4:** Factors influencing weight change variations among HIV/AIDS patients on ART enrolled in the ART Clinic at Hawassa University Comprehensive Specialized Hospital in March 2021.

	Total (n) = 422	TLD (N) = 211	TLE (n) = 211	*P* value
Age in year(mean ± SD)	41.44 ± 10.4	40.28 ± 9.93	42.60 ± 10.75	<0.001
Initial weight in kg (mean ± SD)	57.10 ± 7.86	56.85 ± 7.83	57.38 ± 7.96	<0.001
Current weight in kg (mean ± SD)	60.17 ± 7.27	60.78 ± 7.51	59.57 ± 7.1	<0.001
Weight difference, kg (mean ± SD)	3.07 ± 2.36	3.882 ± .21	2.2559 ± 2.39	<0.001
Initial BMI, kg/m^2^ (mean ± SD)	20.79 ± 1.94	20.62 ± 2	20.96 ± 1.88	<0.001
Current BMI, kg/m2 (mean ± SD)	21.93 ± 1.80	22.06 ± 1.9	21.80 ± 1.71	<0.001
BMI difference, kg/m^2^ (mean ± SD)	1.16 ± .96	1.48 ± .86	.8389 ± .95	<0.001

**Table 5:** A bivariate and multivariate analysis of weight changes among HIV/AIDS patients on ART who enrolled in the ART clinic at Hawassa University Comprehensive Specialized Hospital in March 2021.

S. no.	Variable	Category	Weight change		COR	AOR	*P* value
			<5%	≥5%			
1	Regimens	TLD	74	137	2.055 (1.390, 3.039)^*∗*^	0.892 (0.543, 1.465)	0.652
		TLE	111	100	1	1	

2	Current CD4 (1)	CD4 < 500	130	83	0.228 (0.151, 0.345)^*∗*^	0.315 (0.188, 0.527)^*∗∗*^	0.001
		CD4 ≥ 500	55	154	1	1	

3	Current VL (1)	Viral load ≥1000	53	26	3.258 (1.943, 5.466)^*∗*^	0.776 (0.415, 1.453)	0.428
		Viral load <1000	132	211	1	1	

## Data Availability

Additional data can be available from the correspondent author up on reasonable request.

## References

[1] Hill A. W. L., Waters L. (2019). Are new antiretroviral treatments increasing the risks of clinical obesity?. *Journal of Virus Eradication*.

[2] Vitoria M. H. A., Ford N., Doherty M. (2018). The transition to Doultegravir and other new antiretroviral in low- and middle-income countries – what are the issues?. *AIDS*.

[3] WHO (2018). *Update of Recommendations on First- And Second-Line Antiretroviral Regimens, 2019*.

[4] Wong C. G. S., Gange S. J., Justice A. C. (2018). Multimorbidity among persons living with human immunodeficiency virus in the United States. *Clinical Infectious Diseases*.

[5] Lake J. E. C. J., Currier J. S. (2013). Metabolic disease in HIV infection. *The Lancet Infectious Diseases*.

[6] Dooley K. E., Kaplan R., Mwelase N. (2020). Dolutegravir-based antiretroviral therapy. Dolutegravir-based antiretroviral therapy. *Clinical Infectious Diseases*.

[7] Yuh B. T. J., Tate J., Crothers K. (2015). Weight change after antiretroviral therapy and mortality. *Clinical Infectious Diseases*.

[8] Lakey W. C. Y. L., Yancy W., Chow S. C., Hicks C. B. (2013). From wasting to obesity: initial antiretroviral therapy and weight gain in HIV-infected persons. *AIDS Research and Human Retroviruses*.

[9] Koethe J. R. J. C., Lau B., Shepherd B. E. (2016). Rising obesity prevalence and weight gain among adults starting antiretroviral therapy in the United States and Canada. *AIDS Research and Human Retroviruses*.

[10] Koethe J. R. L. M., Limbada M. I., Nyirenda C. K. (2010). Early immunologic response and subsequent survival among malnourished adults receiving antiretroviral therapy in Urban Zambia. *AIDS*.

[11] Koethe J. R. L. A., Lukusa A., Chi B. H. (2010). Association between weight gain and clinical outcomes among malnourished adults initiating antiretroviral therapy in Lusaka, Zambia. *JAIDS Journal of Acquired Immune Deficiency Syndromes*.

[12] Herrin M. T. J., Tate J. P., Butt A. A. (2016). Weight gain and incident diabetes among HIV-infected veterans initiating antiretroviral therapy compared with uninfected individuals. *JAIDS Journal of Acquired Immune Deficiency Syndromes*.

[13] Butt A. A. M. K., McGinnis K., Crystal S. (2009). HIV infection and the risk of diabetes mellitus. *AIDS*.

[14] Isa S. E. O. A., Oche A. O., Kang’ombe A. R. (2016). Human immunodeficiency virus and risk of type 2 diabetes in a large adult cohort in Jos, Nigeria. *Clinical Infectious Diseases*.

[15] Crum-Cianflone N. R. M., Roediger M. P., Headd M. (2010). Increasing rates of obesity among HIV-infected persons during the HIV epidemic. *PLoS One*.

[16] Phillips A. N. V. F., Havlir D., Pozniak A., Kuritzkes D., Wensing A. (2019). Risks and benefits of doultegravir-based antiretroviral drug regimens in sub-saharan africa: a modelling study. *Lancet HIV*.

[17] Federal ministry of Health (FMOH) E (2015). *Update Ethiopia March 2015 HIV/AIDS Progress in 2014*.

[18] Ethiopia FMO (2018). *National Consolidated Guidelines For Comprehensive Hiv Prevention, Care And Treatment*.

[19] Bakal D. R. C. L., Coelho L. E., Clark J. L. (2018). Obesity following ART initiation is common and influenced by both traditional and HIV-/ART-specific risk factors. *Journal of Antimicrobial Chemotherapy*.

[20] Group TNAS (2019). Dolutegravir-based or low-dose efavirenz– based regimen for the treatment of hiv-1. *The New England Journal O F Medicine*.

[21] Hill A., Venter W. F., Delaporte E. Progressive rises in weight and clinical obesity for TAF/FTC/DTG and TDF/FTC/DTG versus TDF/FTC/EFV: ADVANCE and NAMSAL Trials.

[22] Guaraldi G., Calza S., Milic J. (2021). Dolutegravir is not associated with weight gain in antiretroviral therapy experienced geriatric patients living with hiv. *AIDS*.

[23] USA FaDA (2007). Guidance for Industry. Development of products for weight management. https://www.fda.gov/downloads/Drugs/Guidances/ucm071612.pdf.

[24] Macallan D. C. M. M., McNurlan M. A., Calder A. G., Garlick P. J., Griffin G. E. (1995). Whole-body protein turnover from leucine kinetics and the response to nutrition in human immunodeficiency virus infection. *The American Journal of Clinical Nutrition*.

[25] Powanda M. C. B. W., Beisel W. R. (2003). Metabolic effects of infection on protein and energy status. *Journal of Nutrition*.

[26] Melchior J. C. R. G., Raguin G., Bouvet E. (1993). Resting energy expenditure in human immunodeficiency virus-infected patients: comparison between patients with and without secondary infections. *The American Journal of Clinical Nutrition*.

[27] Grunfeld C. P. M., Pang M., Shigenaga J. K., Jensen P., Feingold K. R. (1992). Resting energy expenditure, caloric intake, and short-term weight change in human immunodeficiency virus infection and the acquired immunodeficiency syndrome. *The American Journal of Clinical Nutrition*.

[28] Shevitz A. H., Knox T. A., Roubenoff R., Gorbach S. L., Skolnik P. R. (1999). Elevated resting energy expenditure among HIV-seropositive persons receiving highly active antiretroviral therapy. *AIDS*.

[29] Melchior J. C., Salmon D., Leport C. (1991). Resting energy expenditure is increased in stable, malnourished HIV-infected patients. *The American Journal of Clinical Nutrition*.

[30] Yarasheski K. E., Zachwieja J. J., Crowley J., Horgan M. M., Powderly W. G. (1998). Increased plasma gln and Leu Ra and inappropriately low muscle protein synthesis rate in AIDS wasting. *American Journal of Physiology-Endocrinology and Metabolism*.

[31] Macallan D. C., Noble C., Jebb S. A. (1995). Energy expenditure and wasting in human immunodeficiency virus infection. *New England Journal of Medicine*.

[32] Jamison N. M. T., Carmen B., Peter R., Bryan S., Sally B. (2017). Weight gain in persons with HIV switched from efavirenz-based to integrase strand transfer inhibitor-based regimens. *Journal of Acquired Immune Deficiency Syndromes*.

[33] Phillips A. N., Bansi-Matharu L., Venter F. (2020). Updated assessment of risks and benefits of Dolutegravir versus efavirenz in new antiretroviral treatment initiators in sub-Saharan Africa: modelling to inform treatment guidelines. *The Lancet HIV*.

[34] Kasper D. F. A., Longo D., Braunwald E., Hauser S., Jameson L. (2015). *Harrison’s Principles of Internal Medicine*.

[35] FMOH (2019). *Implementation Manual for DTG Rollout and ART Optimization in Ethiopia*.

